# Lipid Profile after Pharmacologic Discontinuation and Restoration of Menstruation in Women with Endometriosis: A 12-Month Observational Prospective Study

**DOI:** 10.3390/jcm12165430

**Published:** 2023-08-21

**Authors:** Athanasios D. Anastasilakis, Stergios A. Polyzos, Panagiotis A. Vorkas, Athina Gkiomisi, Maria P. Yavropoulou, Martina Rauner, Panagiotis Nikolakopoulos, Stergios Papachatzopoulos, Polyzois Makras, Spyridon Gerou, Lorenz C. Hofbauer, Andrea Palermo, Elena Tsourdi

**Affiliations:** 1 Department of Endocrinology, 424 Military General Hospital, 56429 Thessaloniki, Greece; 2 First Laboratory of Pharmacology, School of Medicine, Aristotle University of Thessaloniki, 54124 Thessaloniki, Greece; spolyzos@auth.gr; 3 School of Cardiovascular and Metabolic Medicine & Sciences, King’s College London, London SE5 9RJ, UK; v_pns@yahoo.gr; 4 Department of Metabolism, Digestion and Reproduction, Faculty of Medicine, Imperial College London, London SW7 2AZ, UK; pnikolakopoulos.gyn@gmail.com; 5 Institute of Applied Biosciences, Centre for Research and Technology Hellas (INAB|CERTH), 57001 Thessaloniki, Greece; 6 Department of Obstetrics and Gynaecology, 424 General Military Hospital, 56429 Thessaloniki, Greece; athgiom@yahoo.gr (A.G.); sterpapacha@yahoo.gr (S.P.); 7 Endocrinology Unit, First Department of Propaedeutic and Internal Medicine, Medical School, National and Kapodistrian University of Athens, 11527 Athens, Greece; myavropoulou@med.uoa.gr; 8 Department of Medicine III and Center for Healthy Aging, Technische Universität Dresden, 01307 Dresden, Germany; martina.rauner@uniklinikum-dresden.de (M.R.); lorenz.hofbauer@uniklinikum-dresden.de (L.C.H.); elena.tsourdi@uniklinikum-dresden.de (E.T.); 9 Department of Endocrinology and Diabetes and Department of Medical Research, 251 Hellenic Air Force & VA General Hospital, 11525 Athens, Greece; 10 Analysis Laboratories, 54623 Thessaloniki, Greece; sgerou@analysi.gr; 11 Unit of Endocrinology and Diabetes, Campus Bio-Medico University, 00128 Rome, Italy; a.palermo@unicampus.it

**Keywords:** menstrual cessation, GnRH analog, goserelin, lipid, lipidomics, endometriosis

## Abstract

The lipid profile is affected following menstrual cessation (MC). We aimed to evaluate the effects of goserelin-induced MC and subsequent menstrual restoration (MR) on lipid metabolism. Premenopausal women with histologically verified endometriosis (n = 15) received goserelin monthly for 6 months (6mο), resulting in MC, and were followed-up for another 6 months after MR (12mο). Serum total cholesterol (TC), triglycerides (TG), high-density lipoprotein cholesterol (HDL-C), low-density lipoprotein cholesterol (LDL-C), apolipoprotein A1 (ApoA1), apolipoprotein B (ApoB), lipoprotein a ([Lp(a)] and lipidomics were measured at baseline, 6mo and 12mo. Shotgun quantitative deep lipidomics were determined at the level of lipid class category, subclass, species, and fatty acyl chain lengths and degree of saturation. TC (*p* = 0.006), LDL-C (*p* = 0.028), HDL-C (*p* = 0.002), and apoA1 (*p* = 0.013) increased during goserelin-induced MC and remained practically unchanged during MR. TG, apoB, and Lp(a) did not change. From the deep lipidomics analysis, multivariate statistical analysis demonstrated profound alterations in lipid species with MC, whereas no statistically valid models could be fitted for the restoration period. In conclusion, GnRH-analog-induced MC alters lipid profiles at various levels, from standard blood lipid and lipoprotein profiles to several lipid species as detected by lipidomics analysis. Changes largely persist for at least 6 m after MR.

## 1. Introduction

In estrogen-dependent diseases, such as endometriosis, reducing estrogen levels with a long-acting gonadotropin-releasing hormone (GnRH) analog is a common treatment strategy [1,2]. The induced hypoestrogenic state relieves symptoms and achieves disease remission. However, at the same time, it may cause undesirable side effects, mimicking the clinical and metabolic alterations observed in menopause. Well-known side effects in this setting include vasomotor symptoms [3,4], headaches, urogenital atrophy, decreased libido, sleep disturbances, weight gain, and bone loss [4,5]. 

In recent years, there has been considerable progress in our understanding of the mechanisms that regulate lipid metabolism and the changes that occur even at a cellular and subcellular level [6]. The discipline of lipidomics involves chemical analysis approaches for the holistic multiplex determination of hundreds of lipid species simultaneously in a complex biological matrix [7]. Mass spectrometry and liquid chromatography coupled to mass spectrometry are now the techniques of choice for quantifying lipid species down to the subclass level. Furthermore, these techniques can provide information on the incorporated fatty acid lengths and unsaturation levels [8]. Overall, lipidomics determination has supported the quest for elucidating lipid metabolism and function and for providing biomarker candidates [9]. In the context of pharmacologically-induced menstrual cessation, studying the lipid changes that take place could provide further insights into the underlying mechanisms by which estrogen deficiency affects lipid metabolism. However, although it is a very exciting and developing new field, we should clarify here that not all findings of lipidomics analysis can be translated into clinical practice, at least not as of yet.

Thus, to test the hypothesis that transient menstrual cessation, induced by a GnRH analog, and the subsequent menstrual restoration could alter the lipidome, we performed a deep lipidomics analysis along with conventional lipid profile analysis (lipoproteins and apolipoproteins) in serum samples from premenopausal women with endometriosis. 

## 2. Patients and Methods

### 2.1. Patients 

This was an additional analysis performed in a subgroup of patients participating in a 12-month observational prospective cohort study of women with endometriosis [5]. Patients were treated for six months with monthly subcutaneous injections of goserelin acetate (3.6 mg) to induce menstrual cessation. Subsequently, patients were monitored for another 6 months after menstrual restoration (starting counting from the first day of the restored menstrual cycle). A detailed description of the initial cohort of 21 patients has been previously published [5]. The present post hoc analysis was performed in a subgroup of 15 randomly selected otherwise healthy premenopausal women, in whom additional, previously unthawed serum samples at baseline, 6 and 12 months were used to evaluate changes in lipid parameters.

### 2.2. Study Protocol

At baseline, a detailed medical history was obtained, weight and height were measured, and body mass index (BMI) was calculated. Morning fasting blood samples were obtained from the participants at baseline (TP1), at 6 months of treatment with goserelin (6 m, TP2), and 6 months after menstrual restoration (12 m, TP3). The study was approved by the ethics committee of the 424 Military General Hospital and registered at clinicaltrials.gov (NCT04203212). All procedures were in accordance with the Declaration of Helsinki and the International Conference on Harmonization for Good Clinical Practice. Written informed consent was obtained from the patients included in this post-hoc analysis.

### 2.3. Assays

Parameters of lipid metabolism, including serum total cholesterol (TC), triglycerides (TG), high-density lipoprotein cholesterol (HDL-C), low-density lipoprotein cholesterol (LDL-C), apolipoprotein A1 (ApoA1), apolipoprotein B (ApoB), and lipoprotein a [(Lp(a)], were measured in one batch at the end of the study by standard laboratory methods. An additional aliquot of each serum sample was analyzed by Lipotype GmbH, Dresden, Germany, using a previously described shotgun deep lipidomics approach [10]. Briefly, the addition of internal standards to the samples was followed by an organic liquid-liquid extraction. Samples were analyzed using direct infusion in a Q-Exactive mass spectrometer (Thermo Scientific, Waltham, MA, USA). The resulting data were analyzed using an in-house lipid identification software, as presented elsewhere [10].

### 2.4. Statistical Analysis

#### 2.4.1. Standard Lipid Parameters

The Shapiro–Wilk test was used to assess the normality of distributions, and data are presented as mean ± SEM or number (%), as applicable. We used repeated measures analysis of variance (ANOVA) for the comparisons of lipid and lipoprotein concentrations at different time points within the patient group. The Bonferroni correction was used for multiple pairwise comparisons. All *p*-values were two-sided, and a value of *p* < 0.05 was considered statistically significant. Statistical analysis was performed using SPSS Statistics for Mac, Version 27 (IBM Corporation, Armonk, NY, USA).

#### 2.4.2. Lipidomics Data Processing—Statistical and Pathway Analysis Methods

The identified lipid molecules were quantified and normalized to a lipid subclass specific internal standard. The amounts in pmol of the individual lipid molecules of a given lipid subclass were summed to yield the total amount of the lipid subclass. The amounts of the lipid subclasses were normalized to the total lipid amount, yielding mol% per total lipid. Where the signal was too low for quantitation and a value was missing, this value was converted to 10 times less than the minimum normalized lipid amount of the corresponding lipid subclass.

Normalized lipid amounts (mol% per total lipid) were first analyzed using multivariate statistical analysis methods. For this, the SIMCA (Umetrics) package (v. 14) was employed. Each sample was grouped according to timepoint. Analysis was performed first by principal component analysis (PCA) and followed by orthogonal projection to latent structures—discriminant analysis (OPLS-DA). All data were scaled to unit variance. Furthermore, for valid OPLS-DA fitted models, lipids with a cross-validated Variable Influence on Projection (VIPcv) > 1.5 were forwarded for further univariate statistics. For the discriminant lipids, paired *t*-test *p*-values and log2 fold changes (average values) were further calculated.

The list of discriminant lipids was assigned to their corresponding KEGG ID and imported to pathway analysis tools: Reactome Analysis (Pathway Browser v 3.7; Database Release 78) and LIPEA (analysis date: 15 December 2021).

Correlation analysis was performed by first calculating Pearson (r) correlation coefficients in the R programming language (version 4.1.2). The correlation networks were constructed in Cytoscape (version 3.9.1) and MetScape (version 3.1.3).

## 3. Results

Fifteen premenopausal women with endometriosis (age 33.5 ± 1.6 years, BMI 23.4 ± 0.9 kg/m^2^) were included in the analysis. Two of the participants (13%) were smokers, and seven (47%) had given birth to 1–3 children.

### 3.1. Standard Serum Lipid and Lipoprotein Concentrations

Serum lipid and lipoprotein concentrations are presented in Table 1. TC, LDL-C, HDL-C, and apoA1 increased at month 6. HDL-C and apo-A1 remained essentially unchanged at month 12 compared to month 6, whereas TC and LDL-C at month 12 did not statistically significantly differ from either baseline or month 6. TG, apoB, and Lp(a) concentrations did not statistically change throughout the study.

### 3.2. Multivariate Statistics of Lipidomics Analysis

**Figure 1:**
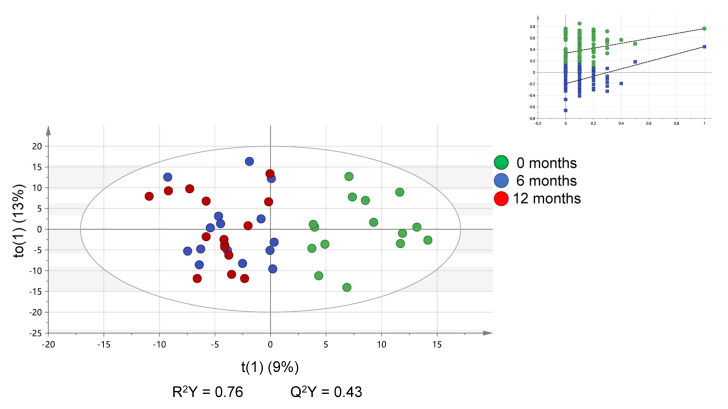
OPLS-DA scores plot of the samples for each of the three timepoints. Variables are scaled to unit variance, and eclipse represents the 95% Hotelling’s T2 (CV-ANOVA *p* = 0.002). The inlet represents results from permutation testing (n = 99) for R2Y (green) and Q2Y (blue).

The PCA-fitted model of the three timepoints, demonstrated a separation of TP1 from TP2 and TP3 in score plots (Figure 1 and Figure S1). However, TP2 and TP3 displayed no separation from each other (Supplementary Figure S1). This was further apparent from an OPLS-DA-fitted model (Figure 1). Pairwise group comparisons using PCA and OPLS-DA also verified this observation. Specifically, the comparison of TP1 against TP2 showed a predictive value (Q2Y) of 0.340 with a model validated via permutation testing and a CV-ANOVA *p*-value of 0.004 (Table 2 and Supplementary Figure S2). On the contrary, the comparison of TP2 to TP3 did not demonstrate any separation between the two timepoints, implying no significant difference between the detected serum lipid species at these timepoints. As expected, TP1 and TP3 also demonstrated a valid and predictive model (Q2Y = 0.420) (Table 2 and Supplementary Figure S3). A summary of all pairwise OPLS-DA fitted model characteristics is presented in Table 2.

### 3.3. Univariate Statistics of Lipid Profiling

**Figure 2:**
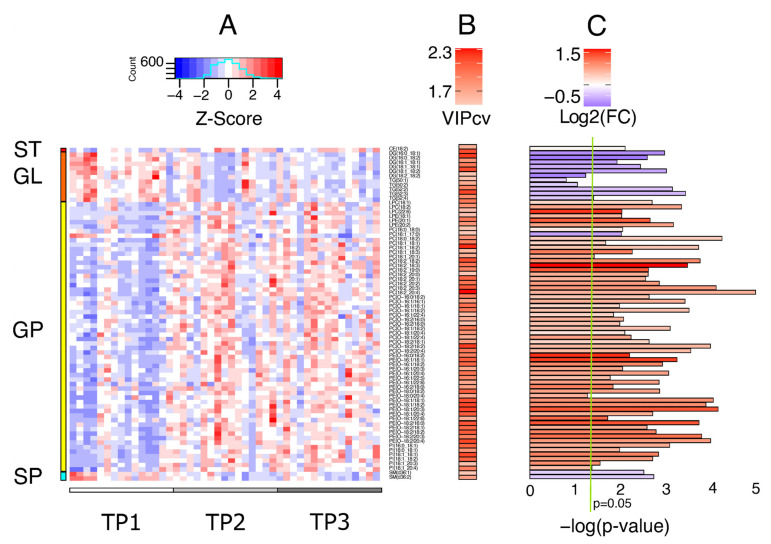
A summative depiction of the lipidomics data and resulting statistics. (**A**). A heatmap of the lipid concentrations plotted as lipid Z-scores. (**B**). A heatmap of the VIPcv (cross-validated variable influence on projection) values (VIPcv > 1.5) obtained by the OPLS-DA fitted model for the comparison of TP1 vs. TP2. (**C**). Bar-plots of the negative base 10 logarithm of the *p*-values (–log(*p*-value) from the paired *t*-test comparison of TP1 vs. TP2. The color-coding represents the base 2 logarithm of the fold changes (log2(FC)) for the same comparison. FC was calculated as the quotient of the average of the values of TP2 divided by the average of TP1. Abbreviations: CE: cholesteryl ester; DG: diacylglycerol; GL: glycerolipids; GP: glycerophospholipids; LPC: monoacylglycerophosphocholines (lysophosphocholine); LPE: monoacylglyceroethanolamine (lysophosphoethanolamine); PC: diacylglycerophosphocholine; PE: diacylglycerophosphoethanolamine; PI: diacylglycerophosphoinositol; SM: sphingomyelin; SP: sphingolipids; ST: sterol lipids; TG: triacylglycerol.

A total of 74 lipid species demonstrated VIPcv values of more than 1.5 for the TP1 vs. TP2 comparison (Figure 2B and Supplementary Table S1). These lipid moieties were further assessed by univariate statistics. All conducted paired *t*-tests demonstrated a *p*-value of <0.05, except for three glycerolipids (one diacylglycerol [DG] and two triacylglycerols [TGs]), as well as one glycerophospholipid, one ether-glycerophosphoethanolamine (O-PE) (Figure 2C and Supplementary Table S1). The log2 fold change values were in the range of 1.7 to −0.99 (Figure 2C and Supplementary Table S1).

### 3.4. Altered Lipid Species

From glycerolipids, a total of 6 DGs and 5 TGs were significantly altered. They all displayed lower concentrations in TP2 as compared to TP1 (Figure 2 and Supplementary Table S1). All DGs were esterified with 16C and 18C fatty acyl chains (FACs) with 0 to 2 double bonds. The TGs were not structurally elucidated to the level of FACs. Their bulk carbons were 50 or 52, with double bonds ranging between 1 and 4. From glycerophospholipids (GPs), 15 glycerophosphocholines (PCs), 3 lysoPCs, 13 ether-PCs, 20 O-PEs, 3 lysoPEs, and 6 glycerophosphoinositols (PIs) were significantly altered. All the GP were increased in TP2 serum samples. This, with the exception of PC(16:1_17:0) which demonstrated a 65% decrease in TP2. For the sphingolipids, two sphingomyelins (SMs) had a significantly decreased normalized concentration. Lastly, a cholesteryl ester (CE) and specifically CE(18:2) was increased in TP2, however at a very low proportion (9%).

### 3.5. Pathway Analysis

Pathway analysis using the REACTOME tool demonstrated predominantly lipoprotein-related pathways (Supplementary Table S2). On the other hand, the LIPEA platform resulted in the two top hits being related to GP metabolism and choline, with the third most significant pathway involving fat digestion and absorption (Supplementary Table S3).

### 3.6. Correlation Analysis

**Figure 3:**
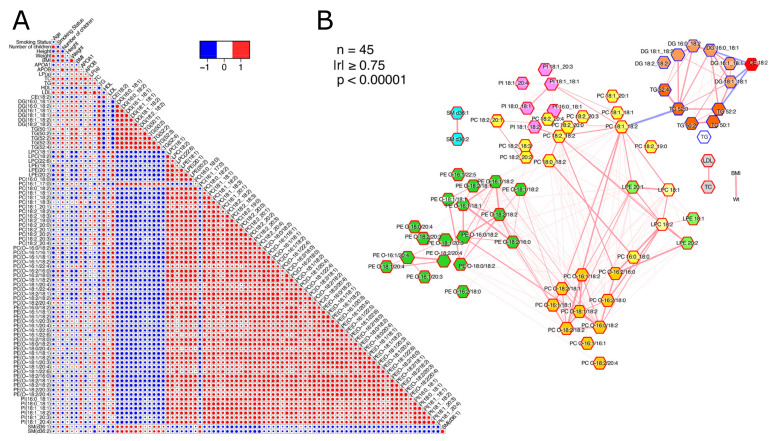
(**A**). Heatmap depicting Pearson correlation coefficients of demographics and lipid moieties. (**B**). Network of Pearson correlation coefficients. Nodes represent variables, and edge thickness and color represent correlation coefficients.

From the correlation analysis (Figure 3A and Supplementary Table S4), the two major trends observed in lipid moieties were associated with lipid categories and subclasses. On the one hand, the SMs, DGs, and TGs, and on the other hand, the CEs and GPs. In the latter category, PC(16:1_17:0) demonstrated a profound exception in the GP category, with an opposite trend. The highest interclass associations were presented between the CE to DGs and TGs, and TG(52:3) to PC(18:1_18:2).

## 4. Discussion

In the present study, we aimed to investigate the effects of transient pharmacological menstrual cessation on lipid metabolism. We used a model of transient, iatrogenic “pseudo-menopause”, which included a relatively homogenous group of otherwise healthy premenopausal women without co-morbidities, and therefore presents several advantages in the study of the effects of estrogen on lipid metabolism by avoiding important confounders that could affect our findings and their interpretation. The longitudinal mode of our experimental design, which subsequently allows for each patient to function as their own control, reduces variation in our sample set. Furthermore, we opted for a multivariate statistical analysis, which is capable of handling data with large numbers of variables, as is the case with lipidomics, and in a relatively small group of observations (patients). These features of our experimental design provide further impact on our findings. To the best of our knowledge, the present study is the first to evaluate lipidomics during pharmacological menstrual cessation and restoration.

Overall, we report here significant changes in both standard blood lipid parameters and lipid species with menstrual cessation. Importantly, alterations in lipid concentrations were not fully reversed 6 months after menstrual restoration. This finding is in concordance with observations involving bone metabolism in the same setting, with bone loss during the halt of menstruation not being fully reversed after its restoration [5].

The increases in TC and LDL-C that we detected have been previously reported both following menopause [11] and after menstrual cessation in women with endometriosis [3,4,12,13,14]. It is not clear whether the observed concomitant increases in HDL-C and apoA1 could play a compensatory role with regard to CV risk. Similar increases in HDL-C and even apo-A1 after GnRH analog-induced menstrual cessation have been reported by other studies [4,13,14,15]. In contrast, no change in HDL-C levels has been reported in women subjected to oophorectomy [16] or transitioning to menopause [17,18]. A loss of the protective effect of HDL-C after menopause has also been suggested [19], but it is uncertain if the same applies to GnRH analog-induced menstrual cessation. Despite the clear increases in TC and LDL-C, we failed to identify a similar increase in apoB, in contrast to a previous study [13]. Although the lack of a similar pattern between ApoB and LDL-C might have been attributed to the small sample size of our study, it must be noted that ApoB and TC or LDL-C were well-correlated (r = 0.52 and 0.65, respectively). Nonetheless, a definite conclusion for the different pattern between Apo-B and LDL-C requires mechanistic studies, which are beyond the aim and design of this study. Furthermore, both TC and LDL-C concentrations at month 12 did not statistically differ from either baseline or month 6. However, since the TC and LDL-C levels at month 6 were increased compared to baseline but at month 12 were not statistically different from those at month 6 (Table 1), we could hypothesize that a 6-month period is not adequate for their restoration. Still, this requires a longer-term study and a larger sample size for more conclusive observations.

A profound change in neutral lipid (glycerol lipids and cholesteryl esters) concentrations was observed following menstrual cessation. Firstly, TGs and DGs demonstrated a reduction in serum. Importantly, these two lipid subclasses have been associated with a higher CV risk in previous [20] and more recent cohort studies [21]. In the latter study, DGs were further highly associated with type 2 diabetes mellitus, though certain TG species appeared to follow inverse trends depending on the incorporated fatty acyl-chains. The highlighted pathway of fat digestion and absorption from pathway analysis could represent a potential cause for this change. Furthermore, although statistically significant, CE(18:2) only displayed a mild increase (9%) following menstrual cessation. The CE species have been associated with CV risk [20,21] and CE(18:2) in particular has been identified in high abundance in atherosclerotic plaques [22]. Corroborating this, in our study, some of the GP species with highly statistically significant alterations following menstrual cessation were those bound to 18:2 fatty chains. Further GPs demonstrating changes with high statistical significance were O-PEs, which have been associated with intracellular cholesterol transport [23].

The lipid PC species PC (16:1_17:0) was the only GP lipid species that exhibited different trends as compared to the rest of this lipid category and subclass, i.e., it was the only GP that decreased during menstrual cessation. Furthermore, it demonstrated highly significant inverse correlations with GP esterified with the arachidonic acid (20:4) chain (Figure 3), implying a potential involvement in (anti-)inflammatory processes. In the past decade, but also recently, odd-chain lipid species have been associated with a lower risk of CV events [21,24]. The odd-chain fatty acids cannot be synthesized by human enzymes and are believed to be obtained from the diet. Studies have suggested that odd-chain lipids could be products of the process of α-oxidation [24]. Furthermore, an end-product of odd-chain fatty acid β-oxidation is propionic acid. Propionic acid has been associated with CV benefits in several studies [25,26] A relationship between serum levels of odd-chain fatty acids and the gut microbiome has also been suggested [27], along with CVD benefits [26,28]. This, in combination with our findings from pathway analysis indicating putative changes in digestion and absorption, necessitates further investigation. Lastly, another PC incorporating an odd chain, PC(18:2_19:0), did not demonstrate the same trend as PC(16:1_17:0) in our study. However, this PC was also incorporating the 18:2 fatty chain, a chain with an intense opposite trend, which could be responsible for the lack of concordance with the 17:0 chain. Overall, the demonstrated decrease of the 17:0 fatty acid is another indication of a detrimental effect on CV health.

The decrease in SMs is an indication of perturbations in the sphingolipid pathway. The SM species is synthesized from ceramide as a substrate and catalyzed by the sphingomyelin synthase, while it can be hydrolyzed back to ceramide by the enzyme sphingomyelinase. The SMs play a crucial role in the structure of the plasma membrane, although they are considered biologically inactive. However, the ceramide species have an established involvement in cellular apoptosis [29]. Furthermore, serum concentrations of ceramides have been associated with CV risk in several studies [30,31].

It is interesting that all the above-described changes in both lipids and lipoproteins remained 6 months after the restoration of menstruation. This implies a prolonged change in lipid profile, the CV effects of which are unknown. In contrast to our findings, previous studies were reassuring on this subject, reporting a reversal of the lipid changes they had observed [4,13]. We cannot conclude whether differences in the studied populations, the assays used, or the GnRH analog used might have been, at least partly, responsible for this discrepancy between our and other studies. However, as already mentioned, the changes we observed at any altered lipid measurement persisted in the restoration period.

It is uncertain if the changes we found in premenopausal women in whom menstruation was ceased pharmacologically can be extrapolated to women transitioning to menopause. As mentioned above, it has been suggested that there are differences between the effects of pharmacologically induced menopause and oophorectomy on lipoprotein metabolism [16].

Our study has potential limitations, including the lack of a control group, the small sample size, which however was adequate to reveal significant changes in several of the parameters evaluated, and the relatively short duration of follow-up after treatment discontinuation. Key strengths of our study include the homogenous and clinically well-characterized group of premenopausal women without co-morbidities, which may minimize residual confounding.

In conclusion, our findings suggest that pharmacological menstrual cessation significantly alters lipid metabolism, and the changes persist at least for 6 months after menstrual restoration. A wealth of changes implies a shift in CV risk, although this remains to be further investigated.

## Figures and Tables

**Table 1:** Lipid and lipoprotein concentrations of the group at the three timepoints (TP) of the study.

Parameter	TP1 (Baseline)	TP2 (Month 6)	TP3 (Month 12)	*p*-Value for Trend
Total cholesterol (mg/dL)	206 ± 6	243 ± 11 ^a^	233 ± 9	0.006
Triglycerides (mg/dL)	118 ± 11	113 ± 19	93 ± 8	0.185
HDL-C (mg/dL)	48 ± 5	60 ± 6 ^a^	61 ± 4 ^a^	0.002
LDL-C (mg/dL)	134 ± 6	158 ± 11 ^a^	152 ± 9	0.028
Lp(a) (mg/dL)	11.8 ± 2.1	17.0 ± 3.2	14.3 ± 3.1	0.056
ApoA1 (mg/dL)	180 ± 9	222 ± 12 ^a^	218 ± 12 ^a^	0.013
ApoB (mg/dL)	101 ± 7	96 ± 9	100 ± 5	0.693

Repeated measures analysis of variance (ANOVA). ^a^: *p* < 0.05 compared with baseline (Bonferroni correction for multiple pairwise comparisons).

**Table 2:** Summary of OPLS-DA (supervised) model characteristics.

TP Comparison	No. of Components (Predictive + Orthogonal)	N	R^2^X	R^2^Y	Q^2^Y	CV-ANOVA *p*-Value
TP1 vs. TP2	1 + 0	30	0.153	0.599	0.340	0.004
TP2 vs. TP3	1 + 5	30	0.475	0.999	−0.100	1
TP1 vs. TP3	1 + 0	30	0.165	0.632	0.420	0.0006

TP: Timepoint; N: Number of observations included in the model; R2X: Variance of the data (X matrix) explained by the model; R2Y: Variance of the data, explained by the groups (Y matrix); Q2Y: Predictive value of the model; CV-ANOVA: cross-validation ANOVA testing (of residual values from cross-validation testing).

## Data Availability

All datasets generated during and/or analyzed during the current study are not publicly available but can be provided by the corresponding author on reasonable request.

## Supplementary Materials

The following supporting information can be downloaded at: https://www.mdpi.com/article/10.3390/jcm12165430/s1, Figure S1: PCA scores plot depicting the samples for each of the three timepoints. Variables are scaled to unit variance and eclipse represents the 95% Hotelling’s T2; Figure S2: OPLS-DA scores plot of the samples for timepoints 1 Vs 2. Variables are scaled to unit variance (CV-ANOVA *p* = 0.004). Inlet represents permutation testing (n = 99) for R2Y (green) and Q2Y (blue); Figure S3: OPLS-DA scores plot of the samples for timepoints 1 Vs 3. Variables are scaled to unit variance (CV-ANOVA *p* = 0.0006). Inlet represents permutation testing (n = 99) for R2Y (green) and Q2Y (blue); Table S1: Statistically significant lipid species in the comparison of baseline (TP1) and at 6 months of treatment (TP2); Table S2: Top 25 pathway as enriched using the REACTOME analysis tool, sorted by *p*-value; Table S3: Top pathways presented with the highest significance using the LIPEA lipid enrichment analysis tool; Table S4: Pearson pairwise correlation coefficients matrix of demographics and lipid moieties.
