# Supplementary material for: Lipid Profile after Pharmacologic Discontinuation and Restoration of Menstruation in Women with Endometriosis: A 12-Month Observational Prospective Study
Source: J Clin Med. 2023 Aug 21;12(16):5430. doi: 10.3390/jcm12165430 (PMC10455875; doi:10.3390/jcm12165430)
Supplement: Supplementary file 1 [file jcm-12-05430-s001.zip › JCM_lipidomics_Supplemental Figure S1.pptx]

## Slide 1
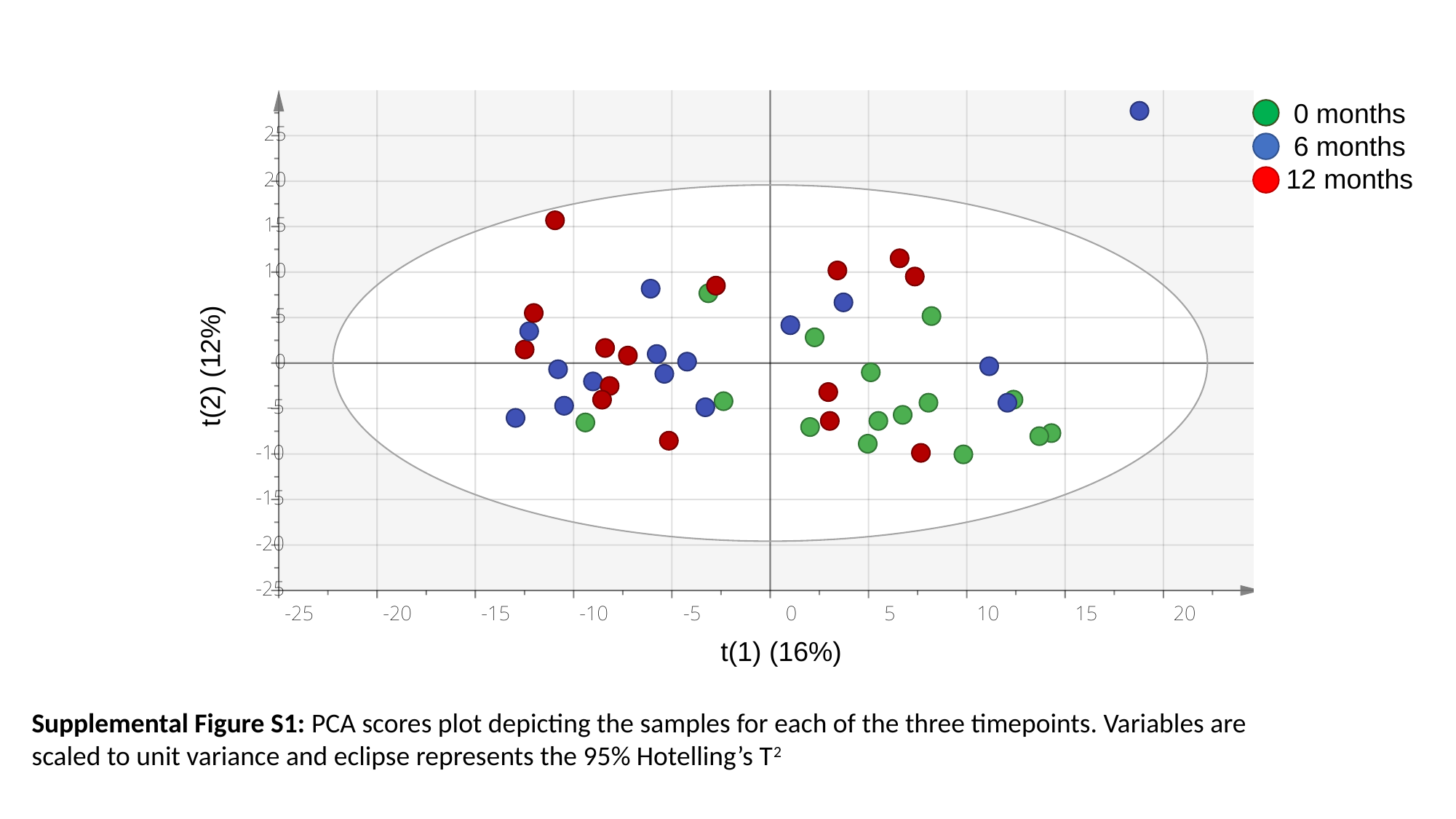

0 months
 6 months
12 months
t(2) (12%)
t(1) (16%)
Supplemental Figure S1: PCA scores plot depicting the samples for each of the three timepoints. Variables are scaled to unit variance and eclipse represents the 95% Hotelling’s T2
