# Supplementary material for: Lipid Profile after Pharmacologic Discontinuation and Restoration of Menstruation in Women with Endometriosis: A 12-Month Observational Prospective Study
Source: J Clin Med. 2023 Aug 21;12(16):5430. doi: 10.3390/jcm12165430 (PMC10455875; doi:10.3390/jcm12165430)
Supplement: Supplementary file 1 [file jcm-12-05430-s001.zip › JCM_lipidomics_Supplemental Figure S2.pptx]

## Slide 1
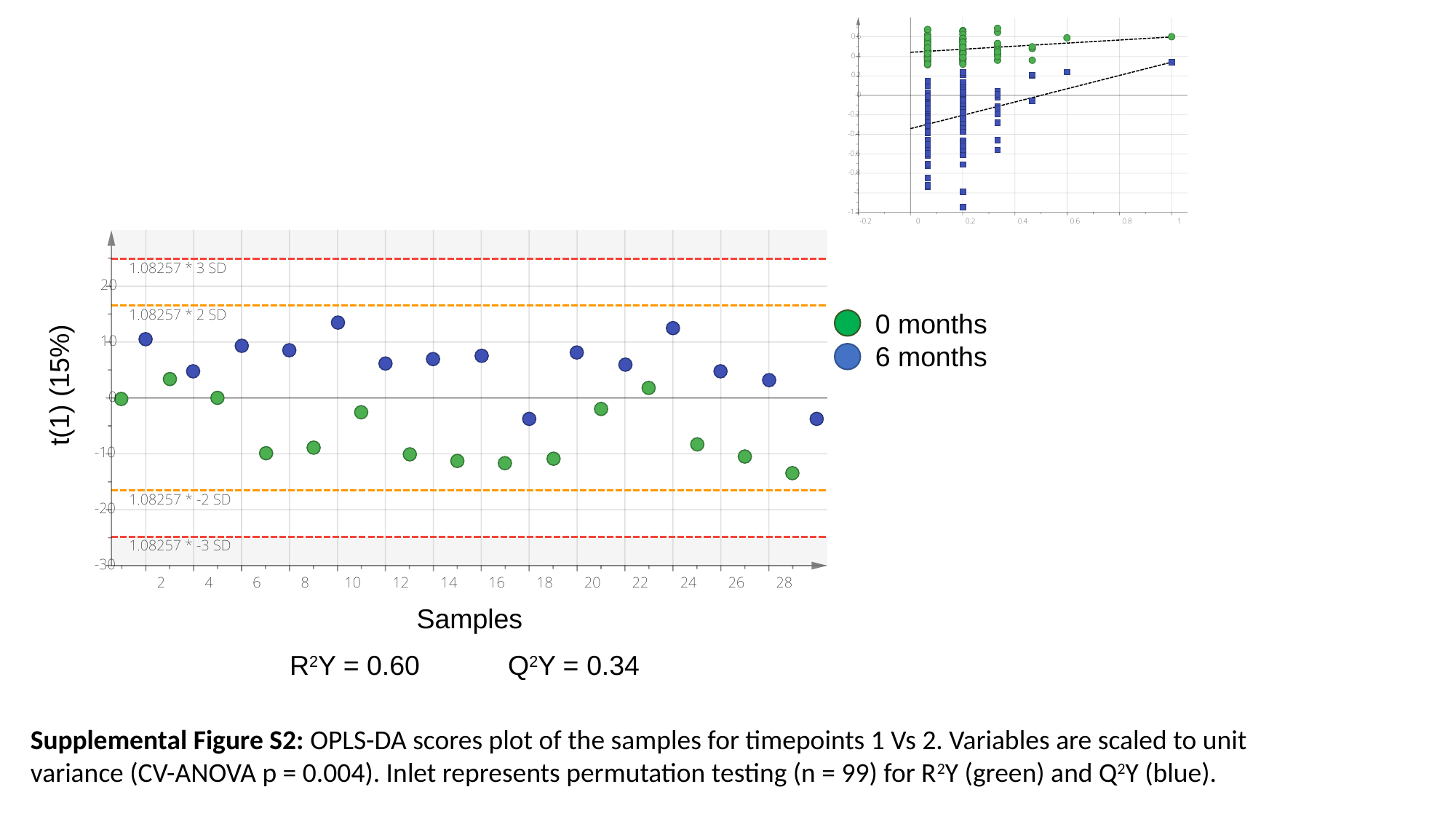

0 months
 6 months
t(1) (15%)
Samples
R2Y = 0.60	Q2Y = 0.34
Supplemental Figure S2: OPLS-DA scores plot of the samples for timepoints 1 Vs 2. Variables are scaled to unit variance (CV-ANOVA p = 0.004). Inlet represents permutation testing (n = 99) for R2Y (green) and Q2Y (blue).
