# Supplementary material for: Lipid Profile after Pharmacologic Discontinuation and Restoration of Menstruation in Women with Endometriosis: A 12-Month Observational Prospective Study
Source: J Clin Med. 2023 Aug 21;12(16):5430. doi: 10.3390/jcm12165430 (PMC10455875; doi:10.3390/jcm12165430)
Supplement: Supplementary file 1 [file jcm-12-05430-s001.zip › JCM_lipidomics_Supplemental Table S1.docx]

**Supplemental Table S1:** Statistically significant lipid species in the comparison of baseline (TP1) and at 6 months of treatment (TP2).

| **Lipid name** | **Structural category** | **VIPcv** | **paired t-test**  **p-value**  **(TP1-TP2)** | **Log2(FC)** |
| --- | --- | --- | --- | --- |
| CE(18:2) | ST | 1.65 | 0.00804 | 0.09 |
| DG(16:0_18:1) | GL | 2.15 | 0.00109 | -0.80 |
| DG(16:0_18:2) | GL | 2.22 | 0.00262 | -0.99 |
| DG(16:1_18:1) | GL | 1.80 | 0.01212 | -0.67 |
| DG(18:1_18:1) | GL | 1.94 | 0.00371 | -0.57 |
| DG(18:1_18:2) | GL | 2.16 | 0.00099 | -0.63 |
| DG(18:2_18:2) | GL | 1.53 | 0.05917 | -0.88 |
| TG(50:1) | GL | 1.55 | 0.15822 | -0.33 |
| TG(50:2) | GL | 1.69 | 0.08975 | -0.34 |
| TG(52:2) | GL | 1.90 | 0.00073 | -0.43 |
| TG(52:3) | GL | 2.18 | 0.00038 | -0.49 |
| TG(52:4) | GL | 1.85 | 0.03963 | -0.38 |
| LPC(18:1) | GP | 1.96 | 0.00205 | 0.37 |
| LPC(18:2) | GP | 1.82 | 0.00046 | 0.76 |
| LPC(22:6) | GP | 1.58 | 0.00946 | 1.53 |
| LPE(18:1) | GP | 1.56 | 0.00958 | 0.63 |
| LPE(20:1) | GP | 1.96 | 0.00227 | 1.32 |
| LPE(20:2) | GP | 1.80 | 0.00070 | 0.80 |
| PC(16:0_18:0) | GP | 1.54 | 0.00909 | 0.19 |
| PC(16:1_17:0) | GP | 1.50 | 0.00974 | -0.65 |
| PC(18:0_18:2) | GP | 1.81 | 0.00006 | 0.43 |
| PC(18:1_18:1) | GP | 1.90 | 0.02142 | 0.38 |
| PC(18:1_18:2) | GP | 2.26 | 0.00019 | 0.50 |
| PC(18:1_18:3) | GP | 1.60 | 0.00560 | 1.12 |
| PC(18:1_20:1) | GP | 1.69 | 0.03844 | 0.66 |
| PC(18:2_18:2) | GP | 2.16 | 0.00018 | 0.98 |
| PC(18:2_18:3) | GP | 1.99 | 0.00034 | 1.66 |
| PC(18:2_19:0) | GP | 1.88 | 0.00247 | 1.34 |
| PC(18:2_20:0) | GP | 1.53 | 0.00250 | 0.70 |
| PC(18:2_20:1) | GP | 2.05 | 0.00285 | 0.63 |
| PC(18:2_20:2) | GP | 1.97 | 0.00143 | 0.85 |
| PC(18:2_20:3) | GP | 2.10 | 0.00008 | 0.86 |
| PC(18:2_20:4) | GP | 2.31 | 0.00001 | 0.66 |
| PC(O-16:0/18:2) | GP | 1.91 | 0.00239 | 0.49 |
| PC(O-16:1/16:1) | GP | 1.75 | 0.00039 | 0.68 |
| PC(O-16:1/18:1) | GP | 2.00 | 0.01071 | 0.36 |
| PC(O-16:1/18:2) | GP | 1.96 | 0.00032 | 0.54 |
| PC(O-16:1/22:4) | GP | 1.59 | 0.01443 | 0.38 |
| PC(O-16:2/16:0) | GP | 1.68 | 0.00869 | 0.67 |
| PC(O-16:2/18:0) | GP | 1.55 | 0.01057 | 0.53 |
| PC(O-18:1/18:2) | GP | 1.97 | 0.00082 | 0.53 |
| PC(O-18:1/20:4) | GP | 1.82 | 0.00815 | 0.25 |
| PC(O-18:1/22:4) | GP | 1.67 | 0.00595 | 0.81 |
| PC(O-18:2/18:1) | GP | 1.77 | 0.00239 | 0.37 |
| PC(O-18:2/18:2) | GP | 2.21 | 0.00011 | 0.60 |
| PC(O-18:2/20:4) | GP | 1.96 | 0.00029 | 0.58 |
| PE(O-16:0/18:2) | GP | 1.91 | 0.00640 | 1.56 |
| PE(O-16:1/18:1) | GP | 2.09 | 0.00058 | 1.50 |
| PE(O-16:1/18:2) | GP | 2.04 | 0.00121 | 0.99 |
| PE(O-16:1/20:3) | GP | 1.70 | 0.00916 | 0.74 |
| PE(O-16:1/20:4) | GP | 1.98 | 0.00089 | 0.78 |
| PE(O-16:1/22:5) | GP | 1.87 | 0.01711 | 0.53 |
| PE(O-16:1/22:6) | GP | 1.70 | 0.00144 | 0.79 |
| PE(O-16:2/18:0) | GP | 1.73 | 0.01482 | 1.06 |
| PE(O-18:0/18:2) | GP | 2.07 | 0.00140 | 0.79 |
| PE(O-18:0/20:4) | GP | 1.71 | 0.05396 | 0.65 |
| PE(O-18:1/18:1) | GP | 2.14 | 0.00009 | 0.92 |
| PE(O-18:1/18:2) | GP | 2.18 | 0.00013 | 0.93 |
| PE(O-18:1/20:3) | GP | 2.19 | 0.00007 | 1.36 |
| PE(O-18:1/20:4) | GP | 2.07 | 0.00199 | 0.68 |
| PE(O-18:1/22:6) | GP | 1.65 | 0.01936 | 1.24 |
| PE(O-18:2/16:0) | GP | 2.11 | 0.00019 | 1.37 |
| PE(O-18:2/18:1) | GP | 2.03 | 0.00265 | 0.67 |
| PE(O-18:2/18:2) | GP | 2.08 | 0.00168 | 1.04 |
| PE(O-18:2/20:3) | GP | 1.91 | 0.00017 | 1.17 |
| PE(O-18:2/20:4) | GP | 2.18 | 0.00011 | 0.87 |
| PI(16:0_18:1) | GP | 2.05 | 0.00084 | 0.69 |
| PI(18:0_18:1) | GP | 1.82 | 0.01062 | 0.63 |
| PI(18:1_18:1) | GP | 2.16 | 0.00148 | 1.04 |
| PI(18:1_18:2) | GP | 2.04 | 0.00199 | 0.81 |
| PI(18:1_20:3) | GP | 1.55 | 0.02854 | 0.94 |
| PI(18:1_20:4) | GP | 1.91 | 0.04321 | 0.55 |
| SM(d36:1) | SL | 1.52 | 0.00312 | -0.32 |
| SM(d36:2) | SL | 1.73 | 0.00189 | -0.36 |

ST: Sterol lipids

GL: Glycerolipids

GP: Glycerophospholipids

SP: Sphingolipids

CE: Cholesteryl ester

DG: Diacylglycerol

TG: Triacylglycerol

LPC: Monoacylglycerophosphocholines (lysophosphocholine)

LPE: Monoacylglyceroethanolamine (lysophosphoethanolamine)

PC: Diacylglycerophosphocholine

PE: Diacylglycerophosphoethanolamine

PI: Diacylglycerophosphoinositol

SM: Sphingomyelin

VIPcv: Cross-validated Variable Influence of Projection

Log2FC: Base-two logarithm of the fold change (FC); FC was calculated as the quotient of the average of the values of TP2 divided by the average of TP1.
