# Supplementary material for: Lipid Profile after Pharmacologic Discontinuation and Restoration of Menstruation in Women with Endometriosis: A 12-Month Observational Prospective Study
Source: J Clin Med. 2023 Aug 21;12(16):5430. doi: 10.3390/jcm12165430 (PMC10455875; doi:10.3390/jcm12165430)

**Supplemental Table S2:** Top 25 pathway as enriched using the REACTOME analysis tool, sorted by p-value


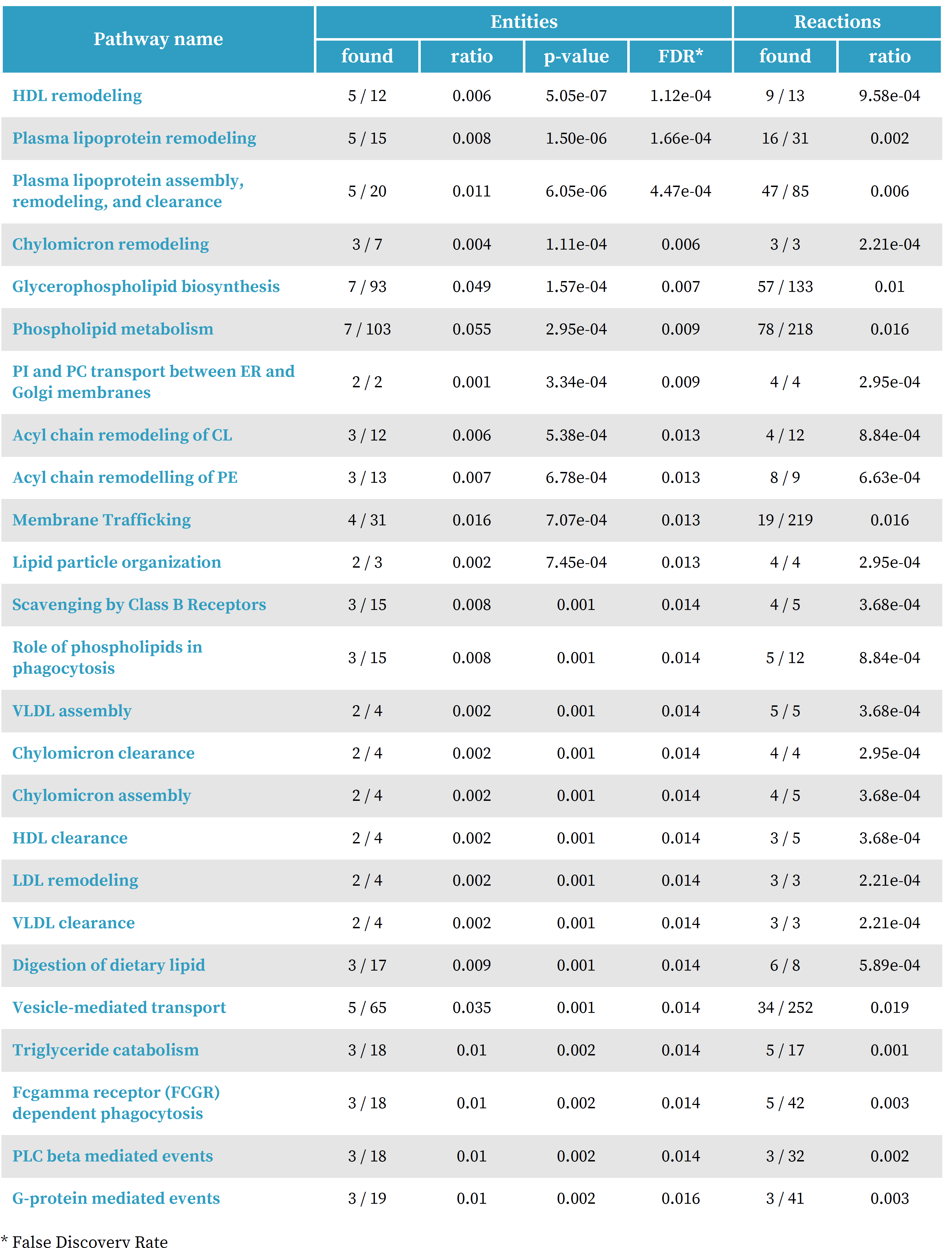

Supplement: Supplementary file 1 [file jcm-12-05430-s001.zip › JCM_lipidomics_Supplemental Table S2.docx]
