# Supplementary material for: Lipid Profile after Pharmacologic Discontinuation and Restoration of Menstruation in Women with Endometriosis: A 12-Month Observational Prospective Study
Source: J Clin Med. 2023 Aug 21;12(16):5430. doi: 10.3390/jcm12165430 (PMC10455875; doi:10.3390/jcm12165430)
Supplement: Supplementary file 1 [file jcm-12-05430-s001.zip › JCM_lipidomics_Supplemental Table S3.docx]

**Supplemental Table S3:** Top pathways presented with the highest significance using the LIPEA lipid enrichment analysis tool.

| **Pathway name** | **Pathway lipids** | **Converted lipids (number)** | **Converted lipids (percentage)** | **Converted lipids (list)** | **p-value** | **Benjamini correction** |
| --- | --- | --- | --- | --- | --- | --- |
| Glycerophospholipid metabolism | 26 | 7 | 29.16666667 | C04438, C04230, C00157, C05973, C00641, C04233, C01194 | 5.56173E-05 | 0.004505003 |
| Choline metabolism in cancer | 5 | 3 | 12.5 | C00165, C04230, C00157 | 0.000755155 | 0.030583759 |
| Fat digestion and absorption | 8 | 3 | 12.5 | C00165, C00422, C02530 | 0.003864226 | 0.10433411 |
| Long-term depression | 3 | 2 | 8.333333333 | C00165, C00641 | 0.00565784 | 0.114571269 |
| Insulin resistance | 4 | 2 | 8.333333333 | C00165, C00422 | 0.011004391 | 0.155118088 |
| Regulation of lipolysis in adipocytes | 6 | 2 | 8.333333333 | C00165, C00422 | 0.026023042 | 0.155118088 |
| Rap1 signaling pathway | 1 | 1 | 4.166666667 | C00165 | 0.04494382 | 0.155118088 |
| NF-kappa B signaling pathway | 1 | 1 | 4.166666667 | C00165 | 0.04494382 | 0.155118088 |
| MAPK signaling pathway | 1 | 1 | 4.166666667 | C00165 | 0.04494382 | 0.155118088 |
| HIF-1 signaling pathway | 1 | 1 | 4.166666667 | C00165 | 0.04494382 | 0.155118088 |
| Adrenergic signaling in cardiomyocytes | 1 | 1 | 4.166666667 | C00165 | 0.04494382 | 0.155118088 |
| Th1 and Th2 cell differentiation | 1 | 1 | 4.166666667 | C00165 | 0.04494382 | 0.155118088 |
| Long-term potentiation | 1 | 1 | 4.166666667 | C00165 | 0.04494382 | 0.155118088 |
| Glutamatergic synapse | 1 | 1 | 4.166666667 | C00165 | 0.04494382 | 0.155118088 |
| Dopaminergic synapse | 1 | 1 | 4.166666667 | C00165 | 0.04494382 | 0.155118088 |
| Circadian entrainment | 1 | 1 | 4.166666667 | C00165 | 0.04494382 | 0.155118088 |
| Melanogenesis | 1 | 1 | 4.166666667 | C00165 | 0.04494382 | 0.155118088 |
| Insulin secretion | 1 | 1 | 4.166666667 | C00165 | 0.04494382 | 0.155118088 |
| Thyroid hormone synthesis | 1 | 1 | 4.166666667 | C00165 | 0.04494382 | 0.155118088 |
| Gastric acid secretion | 1 | 1 | 4.166666667 | C00165 | 0.04494382 | 0.155118088 |
| Salivary secretion | 1 | 1 | 4.166666667 | C00165 | 0.04494382 | 0.155118088 |
| Pancreatic secretion | 1 | 1 | 4.166666667 | C00165 | 0.04494382 | 0.155118088 |
| Retrograde endocannabinoid signaling | 8 | 2 | 8.333333333 | C00165, C00157 | 0.045960915 | 0.155118088 |
| Cholesterol metabolism | 8 | 2 | 8.333333333 | C00422, C02530 | 0.045960915 | 0.155118088 |
